# Sense of Well-Being in Patients with Fibromyalgia: Aerobic Exercise Program in a Mature Forest—A Pilot Study

**DOI:** 10.1155/2015/614783

**Published:** 2015-10-18

**Authors:** Secundino López-Pousa, Glòria Bassets Pagès, Sílvia Monserrat-Vila, Manuel de Gracia Blanco, Jaume Hidalgo Colomé, Josep Garre-Olmo

**Affiliations:** ^1^Neurology Service, Health Care Institute, C/ Dr. Castany, s/n, Salt, 17190 Girona, Spain; ^2^Besalú and Olot Primary Care Team, Catalan Institute of Health, Girona, Spain; ^3^Research Unit, Health Care Institute, Girona, Spain; ^4^Psychology Department, University of Girona, Spain; ^5^Sèlvans Project, Acciónatura, Girona, Spain

## Abstract

*Background and Objective*. Most patients with fibromyalgia benefit from different forms of physical exercise. Studies show that exercise can help restore the body's neurochemical balance and that it triggers a positive emotional state. So, regular exercise can help reduce anxiety, stress, and depression. The aim of this study was to analyze the benefits of moderate aerobic exercise when walking in two types of forests, young and mature, and to assess anxiety, sleep, pain, and well-being in patients with fibromyalgia. Secondary objectives included assessing (i) whether there were differences in temperature, sound, and moisture, (ii) whether there was an improvement in emotional control, and (iii) whether there was an improvement in health (reduction in pain) and in physical and mental relaxation. *Patients and Methods*. A study involving walking through two types of forests (mature and young) was performed. A total of 30 patients were randomly assigned to two groups, mature and young forests. The participants were administered the following tests: the *Spanish version of the Revised Fibromyalgia Impact Questionnaire* (FIQR) at baseline and the end-point of the study, the *State-Trait Anxiety Inventory* (STAI) after each walk, and a series of questions regarding symptomatic evolution. Several physiological parameters were registered. *Results*. FIQR baseline and end-point scores indicated a significant decrease in the symptomatic subscale of the FIQ (SD = 21.7; *z* = −2.4; *p* = 0.041). The within-group analysis revealed that differences were significant with respect to days of intense pain, insomnia, and days of well-being only in the group assigned to the mature forest, not in the group assigned to the young forest. No differences were found with respect to anxiety. *Conclusions*. Although the main aim of this research was not achieved, as the results revealed no differences between the groups in the two forest types, authors could confirm that an aerobic exercise program consisting of walking through a mature forest can provide the subjective perception of having less days of pain and insomnia and more days of wellness, in patients with fibromyalgia.

## 1. Introduction

In recent years, in Japan, the practice of recreational and relaxation activities conducted in forested environments and extensive green spaces for therapeutic purposes has increased considerably. This approach is called forest therapy or “shinrin-yoku” (forest-air bathing and forest-landscape watching/walking) and represents a popular form of natural therapy for the many people looking to reduce stress [1]. Preventive medicine and complementary and alternative medicine have investigated the therapeutic effects of this therapy [2]. Some physiological studies support the hypothesis that walking in the woods has positive effects on the central nervous system, autonomic nervous system, and endocrine system [3–6], increasing the immune response [7, 8], affecting hypertension [9], and positively influencing non-insulin-dependent diabetic patients [10, 11]. Physical activity in forests can have a positive effect on the cardiovascular response of young people [12]. In clinical practice, there already exists some evidence, although heterogeneous, of the positive impact of natural scenery on human health. Similarly, psychological studies indicate that there is a positive emotional response to forest environments because these environments effectively reduce stress and attentional fatigue, help relieve depression, and improve psychological relaxation [13]. Recent studies have indicated that walking through a forest can improve the perception of health conditions and tends to decrease stress in healthy people [13, 14]. Additionally, cognitive and affective improvements have been observed in people with major depressive disorder who performed therapeutic walks in the forest [15]. However, existing evidence on the effects of forest therapy on people's health is limited. A recent systematic revision of randomized clinical studies on the healing and health-improving effects of forest therapy [16] did not find sufficient evidence of such effects due to the poor methodological quality of the trials and the heterogeneous and incomparable protocols employed. Nonetheless, the authors propose a series of strategies and methodological improvements that would make studies of forest therapy viable and would consolidate the limited existing evidence. Following the recommendations proposed by the aforementioned study (e.g., appropriate comparisons in order to explain why forest therapy is better than other types of interventions) [16], the overall objective of this research is to assess the short-term effects of walking through the woods in two natural conditions (primary forest versus secondary forest) on fibromyalgia symptoms and to provide scientific evidence of the results on the health of patients with FM after walking through these woods.

Fibromyalgia (FM) is a rheumatic syndrome of unknown etiology characterized by chronic, diffuse musculoskeletal pain, fatigue, sleep disorders, and morning stiffness and is associated with psychological disorders, mainly anxiety and depression. This condition also exhibits hypersensitivity along with verifiable pain in specific anatomical points [17] and affects 2% to 8% of the population, mostly women [18]. Currently, there is no effective treatment to control FM symptoms, although there is growing evidence of the symptomatic benefit of certain pharmacological treatments (tricyclic antidepressants, inhibitors of serotonin, and norepinephrine and gabapentinoid reuptake modulators) and of certain nonpharmacological therapies (physical exercise, cognitive behavioral therapy, and health education) [18], as well as a balanced diet [19].

The main objective of the study was to assess the short-term effects of walking through the woods in natural conditions on fibromyalgia symptoms and to demonstrate that people with FM performing moderate exercise in therapeutic forests exhibit a significant improvement in their clinical symptoms when compared with the same type of exercise in younger forests. Secondary objectives included assessing (i) whether there are significant differences in temperature, sound, and moisture between mature and young therapeutic forests; (ii) whether people who exercise in therapeutic forests significantly improve emotional control, have a sense of improved health, and observed a reduction in pain; (iii) and whether the therapeutic forest provides patients with greater physical and mental relaxation.

## 2. Patients and Methods

### 2.1. Design and Participants

A randomized single-blind clinical trial of two groups, with individuals 20 to 70 years old who were diagnosed with FM, was designed. People with FM, belonging to the Garrotxa Association of Chronic Fatigue and Fibromyalgia, were invited to participate. People with Chronic Fatigue Syndrome were excluded. Information study documents about the project were sent by mail to the association and potential participants were contacted by telephone to enroll and schedule a meeting prior to starting the study. All patients met the diagnostic criteria of the American College of Rheumatology [17, 20, 21]. A sample size of 15 participants per group (young and mature forest) was selected to provide, with a power of 78.2%, a difference equal to or greater than 30 points in the FIQR scale between both groups, assuming a standard deviation of 30 points and a confidence level of 95%. The study was approved by the Institutional Review Board of the* Institut d'Assistència Sanitària* in Girona. All participants signed informed consent.

### 2.2. Procedure

Participants were assigned to each group using a list of random numbers. Each group took 1.25-kilometer walks in the evenings between 5 and 6 pm during six days. During the walks, participants were accompanied by two nurses trained in interviewing. These nurses were blinded to the type of forest and ensured that the walks were performed in a homogeneous manner. Additionally, the nurses were responsible for managing the data collection notebooks.

### 2.3. Variables and Instruments

Each participant's information regarding age and approximate date of fibromyalgia diagnosis was gathered and self-referential comorbidity was recorded before beginning the study using a standardized questionnaire. Participants' weights and heights were measured on the first and last day of intervention. Blood pressure, heart rate, oxygen saturation, and temperature of the participants were determined at the beginning and end of each walk. In conjunction with the* Spanish version of the Revised Fibromyalgia Impact Questionnaire* (FIQR) [22] and the S*panish version of State-Trait Anxiety Inventory* (STAI) [23] that were administered on the first and last day of intervention, participants completed an ad hoc questionnaire on the symptomatic progression of fibromyalgia during the last 15 days of the trial, specifying the days of generalized discomfort, the days of intense pain, the presence of insomnia, and the number of days during which they experienced well-being. A questionnaire including a self-assessment of the study benefits composed of 9 items with a 0 (negative)–10 (positive) points range was administered the last day of the study. Measures relating to environmental conditions of the forests, such as temperature (in degrees Celsius), luminosity (in lux), noise (in decibels), and atmospheric pressure (in hectopascals), were recorded thirty minutes prior to each session.

### 2.4. Description of the Independent Variable

A young forest is one that presents only first age classes species. Usually, it is a forest with a homogeneous dense or very dense structure and impenetrable undergrowth [24–26].

A mature forest, regardless of its location, urban areas or natural, is one in which the absence of timber exploitation during at least the last 4 or 5 decades has allowed reaching a more advanced and complex structure, with a wider range of age groups, including old trees with a large diameter (usually over 100 years). The closure of the crowns of the trees causes little undergrowth. This composition allows a wide biodiversity and an ecosystem that includes many more types of lichens, fungi, mosses, invertebrates, and their predators, that is, all the flora and fauna in the natural evolution of a forest [24–26].

We refer to a therapeutic forest when the structure of the forest has relatively mature trees, or at least components of maturity (trees), and it is accessible to be visited.

The two forests are located in the Garrotxa Volcanic Zone Natural Park, specifically between Olot and the beech forest in Jordà (Northeast of Girona, Spain). The topography of this area is characterized by rolling hills and small mountains, corresponding to volcanoes of reduced dimensions that emerged approximately 17,000 years ago from lava flow. The forests are mainly composed of wet oak groves of sessile oak (*Quercus robur*), typical of the valley bottoms and quaternary plains that were successively filled by volcanic materials and lacustrine deposits. These forests grow in a middle-European sub-Atlantic climate, in the biogeographic mountain region, specifically the submontane area, and are very rare in the south of the Pyrenees. The walks were performed through flat areas in these woods.

The “Can Serra” mature forest (mature forest group) has many centenarian trees with large and irregular shaped trunk and big roots above the ground. The dominant species is sessile oak, with a harmoniously irregular high mountain structure and a considerable density of old trees with sizable treetops. This area presents rare and very penetrable undergrowth, with sufficient space to accommodate a group of people on a therapeutic walk through the existing trails (Figure 2).

The young forest “Les Llongaines” (young forest group) consists of a more regular and dense woodland with an age range of 5–35 years, without any tree exceeding 50 years. The dominant species is sessile oak, although there is a small sector with beech trees. This is a large open area inhabited by species such as bramble (*Rubus ulmifolius*), hawthorn (*Crataegus monogyna*), or broom (*Cytisus scoparius*). The vegetation is homogeneous, compact, closed, and less penetrable (Figure 3).

### 2.5. Statistical Analysis

We described all the study variables by means of central tendency (mean) and dispersion (standard deviation) measures for quantitative variables. Descriptive data was calculated for the overall sample and stratified according the type of forest (mature and young). In order to assess the effect of forest type in the FIQR scores and in the secondary outcomes we conducted an inferential analysis using a between-group comparison of the differences between baseline and the end of the study scores with the nonparametric Mann-Whitney *U* test. Additionally, we assessed the within-group differences between baseline and the end of the study scores in the FIQR and in the secondary outcomes for each forest group using the Wilcoxon Signed Ranks Test for paired data.

## 3. Results

Initially, 34 participants who were randomized in two groups enrolled in the study. Nonetheless, only 30 of them took part in the research because four dropped out by choice, alleging time incompatibility before the start of the study. All participants were women, and 14 and 16 participants were assigned to the mature forest and to the young forest, respectively. The average age of all participants was 62.3 years (SD = 7.7) with an average weight of 70.7 kg (SD = 14.1) and height of 158.3 cm (SD = 28.2). At baseline systolic blood pressure was of 131.4 mm Hg (SD = 20.4), diastolic blood pressure of 77.2 mm Hg (SD = 9.1), heart rate of 74.4 bpm (SD = 9.2), oxygen saturation of 96.2% (SD = 2.1), and body temperature of 35.4°C (SD = 0.4).  Table 1 presents the baseline characteristics of the participants stratified by group. The comparison of baseline characteristics between the two groups revealed significant differences in systolic blood pressure, which was higher in the participants assigned to the young forest (140.4 versus 121.3; Mann-Whitney* U Z* = −2.55; *p* = 0.009), and oxygen saturation, which was higher in participants assigned to the mature forest (95.3 versus 97.4; Mann-Whitney* U Z* = −2.92; *p* = 0.003).

**Figure 1:**
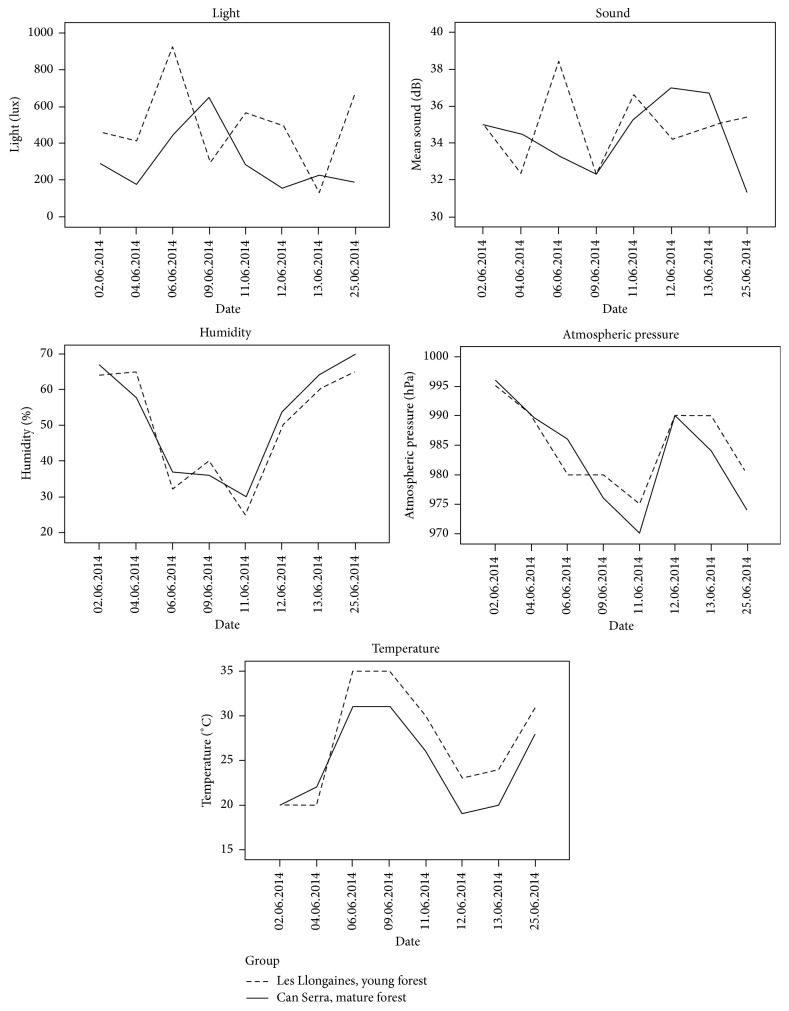
No significant differences were observed between the two forests.

**Figure 2:**
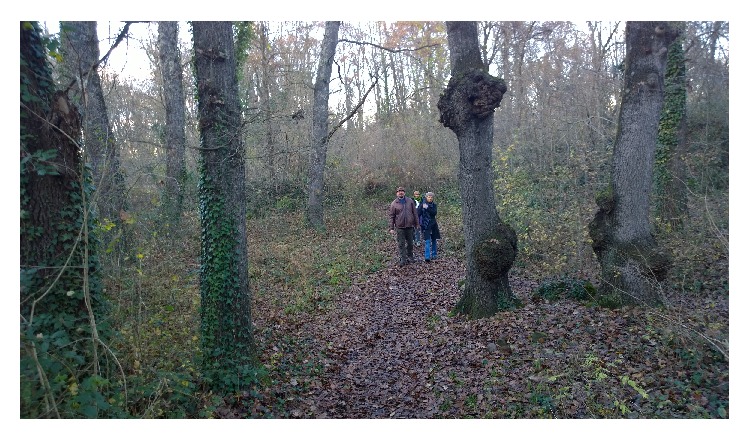
Mature forest “Can Serra.” E(X): 457051/N(Y)4667801.

**Figure 3:**
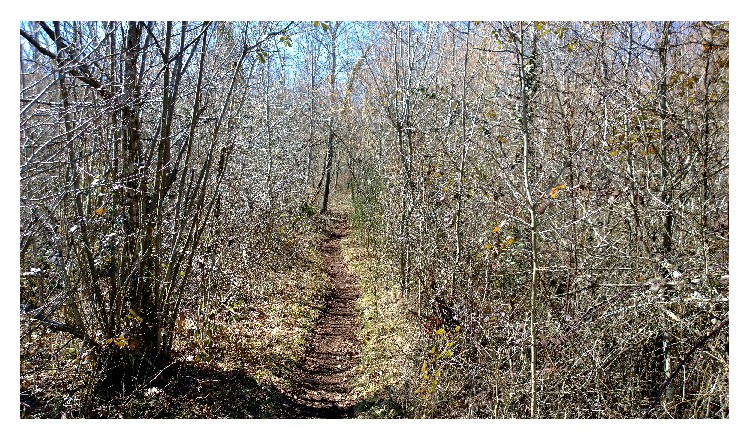
Young forest “Les Llongaines.” E(X): 457216/N(Y)4666983.

With respect to the environmental features during the study, no significant differences were observed between the two forests (light (*p* = 0.083), atmospheric pressure (*p* = 0.673), sound (*p* = 0.656), temperature (*p* = 0.371), and moisture (*p* = 0.816)) (Figure 1).

At baseline, the mean score of all participants in the FIQR scale was 58.7 points (SD = 20.5). The breakdown of the FIQR score on its three subscales was 16.6 points (SD = 6.7) for the functional disability subscale, 10.1 points (SD = 6.6) for the overall impact of the disease subscale, and 32.0 (10.7) points for the subscale of clinical symptoms, without exhibiting statistically significant differences between participants assigned to either group.

In the total sample, the within-group analysis (pre-post) revealed a difference score of 3.8 points (SD = 22.9) for the total FIQR, 1.3 points (SD = 8.0) for the subscale of functional disability, −0.9 points (SD = 7.9) for general impact subscale, and −4.2 points (SD = 11.0) for the clinical symptoms subscale, which was statistically significant (32.0 versus 27.7; Wilcoxon Signed Ranks Test *Z* = −2.04; *p* = 0.41).  Table 2 presents the scores for individual clinical symptoms, where a significant decrease in the severity of symptoms, such as anxiety (Wilcoxon Signed Ranks Test *Z* = −2.86; *p* = 0.004) and tenderness (Wilcoxon Signed Ranks Test *Z* = −2.30; *p* = 0.021), was observed.

The between-group analysis comparing FIQR total and subscale scores revealed no statistically significant differences between the two groups of forests. The group assigned to the mature forest had a score difference in the total FIQR of −6.1 points (SD = 21.3), 0.5 points (SD = 9.0) in the subscale of functional disability, −2.6 points (SD = 6.7) in general impact, and −4.1 points (SD = 11.8) in the clinical symptoms. In the group assigned to the young forest, the difference in the total FIQR was −1.7 points (SD = 24.6), 2.2 points (SD = 7.2) for the subscale of functional disability, 0.6 (SD = 8.6) points for overall impact, and −4.3 points (SD = 11.8) for clinical symptoms.  Table 3 presents the differences in individual symptoms of the FIQR between the two groups.

With respect to anxiety, the mean score for the overall sample of* trait anxiety scale of the STAI* was 4.9 points (SD = 8.3) and 34.9 points for the STAI-state subscale (SD = 9.5). At baseline, no significant differences were observed in any of the STAI subscales among the participants in both groups. State and trait anxiety STAI subscales did not show statistically significant differences between the baseline and the end of the study scores among the groups (differences were not detected within-group analysis nor between-group analysis). Similarly, no differences were found in any of the recorded physiological parameters (blood pressure, heart rate, and body temperature and oxygen saturation) between the start and end of the study.

With respect to subjective assessments on the number of days of perceived well-being/discomfort between baseline and end-point, a decrease in the number of days with symptoms was observed. However this difference was not statistically significant, either for the entire sample or between groups. The within-group analysis revealed that only the mature forest group and not the young forest group exhibited significant differences in the days of intense pain, insomnia, and sense of well-being (Table 4).

Table 5 presents the final scores on the self-assessment of the study benefits. Results indicate that the participants that walked through the mature forest had a better self-assessment of the benefits of the study regarding the degree of relaxation during the walks and will be more prone to recommend this therapy.

## 4. Discussion

The main aim of this research, which was to demonstrate that the clinical benefit of moderate exercise would be superior in mature forests than in younger forests, was not achieved, as the results revealed no differences in the FIQ score between groups. However, participants who walked in the mature forest, unlike those who did so in the young forest, reported significant differences between baseline and final scores with respect to the number of days of intense pain, days of insomnia, and days of well-being. Other controlled studies with FM patients that used different types of activity (moderate exercise, stretching, and educational therapies) reported improvements with respect to pain, functional status, and life quality as well [27, 28]. Our results are very similar to those observed by Arcos-Carmona et al. [29], through a combined program of aerobic exercises and progressive relaxation techniques, given that the benefits of this therapy consisted mainly of improvements in night's rest, pain, and quality of life.

There is a benefit associated with the intervention because the scores in the FIQR subscale for symptoms revealed a global decrease in intensity, specifically pointing to significant differences in anxiety and pain items. Other studies showed similar findings of patient improvement after conducting different types of physical exercises [30–32]. However, the progress observed in our study cannot be attributed to physical exercise in the forest, given that all participants performed the same activity. In our study, participants in the mature forest group, unlike the young forest group, reported improvements in pain, insomnia, and wellness compared with baseline.

Although anxiety is one symptom that typically improves in most studies on exercise and FM, no significant differences in anxiety were found between groups in our study. The lack of response could be attributed to the short duration of the study, which only lasted two weeks. Studies of longer duration, usually more than eight weeks, have obtained better results [33, 34].

Phytoncides are antimicrobial allelochemic volatile organic compounds derived from plants. Some plants give off very active substances which prevent them from rotting or being eaten by some insects and animals or degraded by bacteria and fungi [35]. Due to greater diversity, complexity, and longevity of mature forests, these have more intense and varied phytoncides. In this sense, the immense wealth of volatile components of natural forests structures gives them a huge healing capacity.

When comparing both groups (young/mature forest), the group that walked through the mature forest reported feeling more relaxed than usual, in a statistically significant manner, compared with those who performed the exercise in the young forest. This subjective perception may be related to the alleged benefits of walks through mature woodland. Walking through the forests implies contacting phytoncides produced by trees, as well as enjoying the fresh air, pleasant scenery, and mild climate.

Moreover, the health benefits of activities undertaken in forests can be explained by a better mental and breath control, which involves the recovery of homeostasis [36]. “Natural” stimuli associated with walking in the forests modify oxidative stress and hormonal stress reducing the serum levels of oxidative stress markers such as nitric oxide, malondialdehyde, and catalase, as well as the serum level of cortisol, norepinephrine, and dopamine and significantly increasing serum epinephrine concentrations [37]. This benefit would be higher in patients with (FM) suffering from acute inflammatory state because it could be modified through exercise, inducing a decrease of systemic concentration of IL-8, NA, and cortisol and producing and releasing inflammatory cytokines from monocytic cells [38]; thus the recovery of immune function would be improved [39].

These physiological responses to the environment might interact with each other, leading to positive health outcomes. These mechanisms could be explained with respect to the results observed in other epidemiological studies that have reported positive relationships between the environment and health parameters. At present, there is little evidence on the direct benefits of walking through the woods in reducing chronic pain and fatigue in patients with FM [3].

The study has several limitations. First, due to its exploratory nature, it was underpowered to detect minor differences in the primary efficacy variable, which is the FIQR score. Another limitation, as previously stated, was the short duration of the study length. Perhaps greater differences could have been found if the forest had had therapeutic features closer to those of older forest with more evolved natural dynamics. However, such types of forest are very scarce and valuable and are generally located at a considerable distance from urban areas, which would have prevented the study. Finally, phytoncides were not measured in this study which prevents demonstrating their contribution in the alleviation of symptoms in the FM.

Nonetheless, the results are encouraging and are consistent with those observed in studies of healthy individuals. As previously mentioned, walking in the woods among phytoncide emanations in a pure environment surrounded by landscapes of scenic quality is part of most forest therapy programs. Further research with programs of longer duration, conducted in more mature forests, would clarify the potential benefit of forests in people with FM. Perhaps these exercises could be a complement to existing therapies.

## Figures and Tables

**Table 1:** Characteristics of the participants (mean (SD)).

	Young forest (*n* = 16)	Mature forest (*n* = 14)
Age	60.6 (8.4)	64.4 (6.5)
Weight	73.3 (18.1)	67.8 (6.9)
Height	159.1 (6.6)	157.3 (3.1)
BMI	28.9 (6.9)	27.4 (3.1)
SBP^*∗*^	140.4 (19.0)	121.3 (17.6)
DBP	79.8 (8.6)	77.1 (9.3)
HR	74.7 (10.2)	75.7 (10.2)
SaO_2_^*∗*^	95.3 (2.2)	97.4 (1.6)
BT	35.7 (0.5)	35.9 (0.4)

BMI: body mass index; SBP: systolic blood pressure; DBP: diastolic blood pressure; HR: heart rate; SaO_2_: oxygen saturation; BT: body temperature; ^*∗*^ *p* < 0.05.

**Table 2:** Differences in the FIQR symptom score for all participants between baseline and end-point: within-group (pre-post) analysis.

	Baseline	End-point	Difference
FIQR total score	59.0 (20.6)	55.1 (23.8)	3.8 (22.9)
FIQR functional disability subscale	16.6 (6.6)	17.9 (6.2)	1.3 (8.0)
FIQR general impact subscale	10.1 (6.6)	9.2 (6.2)	0.9 (7.9)
FIQR symptoms subscale	32.0 (10.7)	27.8 (12.4)	4.2 (7.9)
FIQR individual symptoms			
FIQR pain	6.5 (2.3)	5.6 (2.6)	−0.9 (2.3)
FIQR energy	6.7 (3.2)	5.8 (3.1)	−0.9 (4.1)
FIQR stiffness	6.2 (2.9)	5.0 (3.3)	−1.2 (3.1)
FIQR sleep quality	7.7 (2.6)	6.3 (3.3)	−1.4 (3.6)
FIQR depression	5.1 (3.7)	4.7 (3.5)	−0.4 (4.2)
FIQR memory problems	6.1 (3.1)	6.1 (3.3)	0.03 (2.1)
FIQR anxiety	6.3 (3.5)	4.3 (3.6)	−1.9 (3.7)^*∗*^
FIQR tenderness	7.2 (2.8)	5.8 (3.2)	−1.4 (3.0)^*∗*^
FIQR balance problems	5.3 (3.2)	5.2 (3.1)	−0.03 (2.8)
FIQR sensitivity to noise, light, odors, and cold	6.9 (3.3)	6.5 (3.6)	−0.5 (3.7)

^*∗*^ *p* < 0.05.

**Table 3:** Differences in the FIQR symptom score for all participants between baseline and end-point: between-group analysis.

	Young forest (*n* = 16)	Mature forest (*n* = 14)
FIQR total score	−6.1 (21.3)	−1.7 (24.6)
FIQR functional disability subscale	0.5 (9.0)	2.2 (7.2)
FIQR general impact subscale	−2.6 (6.7)	0.6 (8.6)
FIQR symptoms subscale	−4.1 (11.8)	−4.3 (11.8)
FIQR individual symptoms		
FIQR pain	−1.0 (2.6)	−0.7 (2.0)
FIQR energy	−0.7 (3.3)	−1.0 (4.9)
FIQR stiffness	−1.5 (3.4)	−0.9 (2.7)
FIQR sleep quality	−0.9 (3.19)	−1.8 (4.1)
FIQR depression	−0.3 (3.6)	−0.4 (4.9)
FIQR memory problems	−0.1 (2.1)	0.1 (2.0)
FIQR anxiety	−1.0 (3.7)	−2.9 (3.4)
FIQR tenderness	−1.1 (3.0)	−1.6 (3.0)
FIQR balance problems	−0.1 (2.1)	0.1 (3.4)
FIQR sensitivity to noise, light, odors, and cold	−1.6 (2.9)	0.9 (4.0)

**Table 4:** Days of perceived well-being/discomfort at baseline and end-point and differences stratified by the type of forest: within-group (pre-post) analysis.

	Baseline	End-point	Difference
Young forest			
Days of discomfort	12.4 (3.0)	9.4 (5.4)	−3.3 (3.9)
Days of intense pain	8.9 (5.4)	6.1 (5.7)	−2.1 (7.0)
Days of insomnia	7.4 (6.5)	5.6 (6.7)	−1.9 (6.4)
Days with no anxiety	2.9 (5.0)	1.3 (2.1)	−2.1 (6.0)
Days of perceived well-being	1.4 (2.2)	2.9 (4.4)	1.5 (3.0)

Mature forest			
Days of discomfort	8.6 (5.0)	4.8 (6.5)	−3.8 (7.4)
Days of intense pain	7.9 (5.9)	2.5 (4.1)	−5.9 (7.0)^*∗*^
Days of insomnia	7.9 (6.6)	3.7 (5.3)	−4.7 (6.4)^*∗*^
Days with no anxiety	6.1 (5.5)	6.8 (6.8)	1.0 (7.3)
Days of perceived well-being	2.2 (2.5)	7.0 (4.7)	5.0 (4.8)^*∗*^

^*∗*^ *p* < 0.05.

**Table 5:** Assessment at the end of the study (mean (SD)).

	Global	Young forest	Mature forest
I think this therapy was good for me.	8.2 (1.8)	7.8 (1.8)	8.5 (1.8)
During the walks, I have been more relaxed than usual.	8.0 (2.0)	7.1 (2.1)	9.0 (1.4)^*∗*^
I would recommend this therapy to others.	8.9 (2.1)	8.0 (2.7)	9.7 (0.6)^*∗*^
My sleep problems have improved.	5.3 (2.9)	5.6 (2.6)	5.0 (3.3)
I feel less tired.	4.6 (2.8)	4.2 (1.7)	5.1 (3.7)
I feel less pain.	4.7 (2.6)	3.9 (1.8)	5.6 (3.0)
I feel less anxious.	4.5 (3.6)	3.8 (2.0)	5.2 (3.3)
As days went by, I felt greater discomfort.	4.7 (3.6)	3.4 (3.0)	5.9 (3.9)
I would use this therapy again.	95.8%	92.3%	100%

^*∗*^ *p* < 0.05.
